# The impact of social exclusion on experiential sports consumption: the chain mediating roles of loneliness and the need for social connection

**DOI:** 10.3389/fpsyg.2025.1532643

**Published:** 2025-01-29

**Authors:** Chenya Li, Weihua Qu

**Affiliations:** School of Economics and Management, Shanxi University, Taiyuan, Shanxi Province, China

**Keywords:** social exclusion, experiential sports consumption, loneliness, need for social connection, sports consumption

## Abstract

**Introduction:**

With the advancement of the social economy, sports consumption has shifted from traditional material-based purchases to experiential sports consumption, emphasizing psychological engagement and emotional fulfillment. However, the psychological mechanisms behind experiential sports consumption remain underexplored, especially in the context of social exclusion.

**Methods:**

This study is grounded in social exclusion theory, constructing a conceptual model where social exclusion is the independent variable, experiential sports consumption intention is the dependent variable, and loneliness and the need for social connection serve as mediators. A survey was conducted with 415 valid responses, and data were analyzed using structural equation modeling and the Bootstrap method to examine the relationships among the variables.

**Results:**

The findings indicate that social exclusion significantly and positively predicts the intention to engage in experiential sports consumption. It also has a significant positive effect on loneliness and the need for social connection. However, loneliness does not mediate the relationship between social exclusion and experiential sports consumption intention, while the need for social connection does. Furthermore, a chain mediation effect exists between loneliness and the need for social connection in the relationship between social exclusion and experiential sports consumption intention.

**Discussion:**

These results suggest that sports organizations and enterprises should design socially engaging activities that strengthen social connections and alleviate feelings of exclusion. This study enriches the theoretical understanding of social exclusion and its impact on experiential sports consumption, providing practical insights for enhancing social inclusion through sports.

## 1 Introduction

The fast-paced nature of modern society has intensified the prevalence of social exclusion (Pang et al., 2024). This phenomenon often leads to psychological imbalances and prompts individuals to seek relief from this discomfort through specific consumption behaviors (Mead et al., 2011). Against this backdrop, experiential sports consumption may serve as a significant lifestyle choice to mitigate the effects of social exclusion (Matte et al., 2024). Experiential sports consumption is a form of consumption in which individuals directly participate in or engage in sports activities to achieve psychological involvement, emotional satisfaction, and social connection. For example, participating in fitness classes or sports events can help individuals alleviate psychological stress, improve social relationships, and generate lasting wellbeing (Andersen et al., 2019).

Compared to material sports consumption, experiential sports consumption is more unique and irreplaceable (Shank and Lyberger, 2014). It is also less likely to lead to regret among sports consumers (Decrop and Derbaix, 2010) and can provide them with higher and more enduring levels of wellbeing (Armbrecht and Andersson, 2020). The superiority of experiential sports consumption stems from the inherently social nature of sports activities (Yang et al., 2022). Previous research has extensively demonstrated that participation in sports activities can reduce the adverse effects of social exclusion (Dagkas, 2018; Herrmann, 2016), as well as the influence of social exclusion on conspicuous consumption (Lee and Shrum, 2012), green consumption (Guo et al., 2020), compensatory consumption (Rawat et al., 2022), and prosocial behaviors (Knowles and Gardner, 2008). However, the relationship between social exclusion and intention to consume sports remains inconclusive, particularly concerning the lack of in-depth exploration of its psychological mechanisms. For instance, studies have shown that experiential consumption behaviors, such as sports spectatorship, fitness and leisure activities, and sports tourism, can improve social relationships and serve as effective interventions to reduce feelings of social exclusion (Armbrecht and Andersson, 2020; Jiang et al., 2021; Sirgy et al., 2017), the psychological mechanisms and pathways through which social exclusion influences the intention to engage in experiential sports consumption remain unclear. This gap hinders a deeper understanding of its mechanisms and limits the application of related theories in practice.

The sports consumption behaviors triggered by social exclusion reflect individuals' coping strategies to alleviate the threat to their basic psychological needs (Shank and Lyberger, 2014). The Need-Threat Model suggests that social exclusion threatens individuals' basic psychological needs, compelling them to adopt behavioral strategies to alleviate the psychological discomfort caused by these threatened needs (Williams, 2009). For sports consumers, social exclusion may weaken interpersonal relationships, leading to feelings of loneliness (Wang et al., 2022) and prompting individuals to focus more on the need for social connection in sports consumption as a way to cope with this psychological threat (Cacioppo, 2008). Accordingly, based on the Need-Threat Model, this study constructs a relational model involving social exclusion, loneliness, the need for social connection, and experiential sports consumption. It aims to uncover the underlying mechanisms, extend the application of social exclusion theory in sports consumption, and provide empirical evidence and theoretical guidance for relevant practices.

## 2 Literature review

### 2.1 Experiential sports consumption

Based on differences in the purpose of sports consumption, sports consumption behaviors can be divided into two categories: material sports consumption and experiential sports consumption (Matte et al., 2024). Material sports consumption focuses on acquiring tangible sports goods like apparel or equipment, whereas experiential sports consumption emphasizes intangible experiences, such as watching events, fitness activities, or sports training (Baker et al., 2018). Current research generally defines experiential sports as a series of activities that use sports as a medium, involve participation in an experience as the primary form, and aim to promote physical and mental wellbeing.

Experiential sports consumption behaviors, such as watching sports competitions, are often positively associated with psychological states such as a sense of belonging (Jang et al., 2021), social interaction (Wann et al., 2011), self-concept (Bartsch et al., 2018), life satisfaction (Inoue et al., 2017), and wellbeing (Pawlowski et al., 2014). Experiential sports consumption's strong social attributes enhance social relationships by providing opportunities for self-expression and social identification (McDonald and Karg, 2014; Inoue et al., 2017). Even watching sports live streams via online digital platforms can offer viewers a social connection (Waycott et al., 2019), team identity (Yoshida et al., 2018), avenues for expressing opinions (Giglietto et al., 2016), and an escape from reality (Lin et al., 2016) through digital interaction. Under the framework of social identity theory, consumers can reinforce their identity while establishing deeper social connections through participation in symbolic sports activities. For example, in gym settings, consumers interact with others and the environment, enhancing emotional satisfaction and behavioral intentions (Sevilmis et al., 2024).

In terms of self-concept, personal characteristics can influence behavioral preferences. For instance, Baker et al. (2018) demonstrated that consumers' traits affect their satisfaction with sports activities and their likelihood of repeated participation in experiential sports consumption (Baker et al., 2018). Additionally, experiential sports consumption tends to create unique emotional experiences or memories, which, in turn, play a more significant role in shaping self-concept. For example, watching the Paralympic Games fosters empathy and compassion, encouraging prosocial attitudes toward individuals with disabilities (Bartsch et al., 2018).

Regarding wellbeing, existing research indicates that the lasting sense of happiness or satisfaction that sports consumers derive from experiential sports consumption (such as attending live games or participating in sports tourism) is significantly higher than that from material sports consumption (such as purchasing sports apparel or equipment; Armbrecht and Andersson, 2020; Sirgy et al., 2017; Tang and Wang, 2021). There are three main reasons for this phenomenon: (1) experiences are more capable than material wealth of shaping a person's identity and taste (Song et al., 2022); (2) compared to sports products, sports experiences are less likely to trigger negative emotions stemming from social comparison (Carter and Gilovich, 2010); (3) experiential sports consumption is more effective in fostering a sense of social connection (Kumar et al., 2024).

Despite extensive research on experiential sports consumption, the psychological mechanisms underpinning consumer intentions remain underexplored, particularly the roles of social exclusion, loneliness, and social connection.

### 2.2 Social exclusion

Social exclusion is the emotional state in which individuals feel ignored, rejected, or isolated by other individuals or social groups (Williams, 2009). According to the Need-Threat Model, social exclusion, as an unpleasant experience, threatens or undermines four basic psychological needs: the need for belonging, control, self-esteem, and a sense of meaning in life (Freedman et al., 2016; Williams, 2009). It also affects individuals' cognition, emotions, and behaviors in multiple ways (Mourey et al., 2017). Compared to self-determination theory, which emphasizes the role of individuals' positive needs in driving behavior, the Need-Threat Model focuses more on the impairment of psychological needs in adverse situations and their behavioral consequences, making it more suitable for studying the context of social exclusion.

To buffer against the need for threats caused by social exclusion, individuals typically adopt new behaviors or adjust existing ones to cope with the crisis of exclusion, and consumption behavior is one effective strategy for alleviating the effects of social exclusion (Mead et al., 2011). Research suggests that, compared to those not experiencing exclusion, socially excluded individuals are more willing to establish new connections with different social groups (Maner et al., 2007) and are more sensitive to the goal of gaining social acceptance and achieving a sense of belonging (DeWall et al., 2009). Consequently, they are more likely to follow others' opinions in their consumption behaviors, adopting choices that align with group preferences (Williams et al., 2000). They are also more inclined to select products with specific characteristics or symbolic meanings, such as a preference for conspicuous consumption (Lee and Shrum, 2012), green consumption (Guo et al., 2020), anthropomorphic products (Liu et al., 2022), unique products (Wan et al., 2014), and nostalgic products (Kim et al., 2023).

Based on the Need-Threat Model, numerous scholars have begun exploring how individuals respond to social exclusion by engaging in various behaviors to alleviate their threatened psychological needs (Williams, 2009). For example, in terms of brand choice, excluded individuals tend to select brands favored by groups that might accept them, thereby increasing their similarity to the group and re-establishing social connections and a sense of belonging (White and Argo, 2009). Interestingly, in cases where the desired brand is a luxury brand, socially excluded consumers' purchasing intention increases when sales staff exhibit exclusionary behavior (Ward and Dahl, 2014).

Regarding prosocial consumption behaviors, socially excluded individuals may engage in prosocial acts, such as helping others or donating to charity, to attract positive attention and gain others' approval (Knowles and Gardner, 2008), thereby restoring interpersonal belongingness (Lakin and Chartrand, 2003). However, socially excluded individuals whose efficacy needs are threatened may also exhibit negative behavioral responses, such as reducing donations, volunteering, or cooperation with others (Twenge et al., 2007).

In sports studies, it has been further pointed out that leisure sports activities, as a form of social interaction, may be disrupted when individuals experience exclusion or rejection from peers (Collins, 2004). Conversely, empirical studies have shown that sports can enhance peer interaction, improve social relationships, and reduce social exclusion (Herrmann, 2016). However, existing studies mainly focus on general sports participation, with limited exploration of how social exclusion drives experiential sports consumption by threatening basic psychological needs. This study aims to reveal the psychological mechanisms behind this process further.

### 2.3 Loneliness

Loneliness is a negative subjective emotional experience that arises when individuals feel their social or emotional relationships are unmet or fall short of expectations (Trucharte et al., 2023). Loneliness is prevalent across all age groups (Matthews et al., 2022), and individuals with varying levels of loneliness exhibit different behavioral and psychological responses toward social interactions and social needs (Berezan et al., 2020).

Loneliness is a prolonged emotional stressor, with various factors contributing to its onset. Some of the main perspectives are as follows: From a social psychology perspective, loneliness tends to arise when trusted interpersonal relationships, such as family, friendship, or romantic connections, fail to meet an individual's expected level of social interaction (Pittman and Reich, 2016). Hofman et al. (2022) highlighted that individuals with characteristics such as low income, low education, unemployment, single status, living alone, immigrant background, poor health, or chronic illness are more prone to experiencing higher levels of loneliness (Hofman et al., 2022). Andrew and Meeks (2018) pointed out that an individual's perceived sense of personal control significantly negatively impacts the intensity of their loneliness (Andrew and Meeks, 2018). Social comparison is another critical factor; upward social comparison (comparing oneself to those better off) tends to increase loneliness, while downward social comparison (comparing oneself to those worse off) can alleviate it (Arnold et al., 2021).

Most literature emphasizes that loneliness is an aversive psychological experience typically associated with adverse outcomes, such as weakened immune function (Pressman et al., 2005), a sense of loss of control (Stavrova et al., 2022), and diminished wellbeing (Becker et al., 2021). Loneliness has a complex impact with potential positive value. According to evolutionary theory, it heightens self-focus and threat sensitivity (Hofman et al., 2022). Under social exclusion, individuals show greater interest in forming new friendships, more positive impressions of others, and an increased willingness to share rewards (Maner et al., 2007). Moreover, individuals may adopt specific consumption behaviors to cope with loneliness. Research on online consumption has found that products with many positive reviews only appeal to non-lonely consumers. In contrast, such reviews might be counterproductive for consumers experiencing high levels of loneliness due to their lack of social connection (Wang et al., 2012).

Moreover, studies have shown that individuals with high levels of loneliness, due to a lack of real-life social connections, tend to seek symbolic connections through product consumption. For instance, they prefer second-hand products, which can establish a symbolic link between the current and previous owners (Huang and Fishbach, 2021). In the realm of sports studies, it has been noted that sports activities can serve as effective interventions to improve wellbeing and alleviate loneliness. Attending live sports events, for example, can significantly enhance sports consumers' sense of wellbeing and reduce feelings of loneliness (Keyes et al., 2023). Furthermore, Oshimi et al. (2023) found that sports activities (including moderate- and high-intensity activities) and passive sports participation (such as watching sports events) indirectly reduce loneliness by enhancing individuals' eudaimonic wellbeing (Oshimi et al., 2023). However, existing research has yet to thoroughly examine the specific mechanisms of loneliness in sports consumption, mainly whether loneliness influences consumers' preferences and choices in sports consumption. This presents an important direction for future research.

### 2.4 Need for social connection

Social connection refers to an individual's subjective perception of the intimacy level in their interpersonal relationships (Maner et al., 2007), while the need for social connection reflects the desire to fulfill feelings of companionship, belonging, and connection through interactions with others or being part of a specific group (Vella-Brodrick et al., 2023). Individuals with a high need for social connection are likelier to engage in group activities, while those with a low need tend to avoid social opportunities (Lee et al., 2001).

The need for social connection is often directly influenced by an individual's subjective perceptions. When these perceptions are disrupted or diminished, individuals may experience a sense of social disconnection (Cacioppo, 2008). Specifically, social disconnection is closely linked to a range of psychological states, including social anxiety, low self-esteem, low agreeableness, and poor social skills. When individuals perceive social disconnection, they will likely experience a heightened need for social connection (Lim et al., 2016). Additionally, research has pointed out that social exclusion can significantly affect an individual's need for social connection. In such contexts, the basic psychological needs of excluded individuals, especially their need for social connection, may be substantially undermined.

Individuals may engage in compensatory behaviors to alleviate the distress caused by unmet social connection needs. For instance, they may actively seek out social connections, imagine essential relationships, or increase their attention to social cues in their environment. Moreover, individuals may turn to specific consumption behaviors for compensation. For example, they might purchase anthropomorphic brands or products and form connections with these human-like brands or items to satisfy their need for social connection (Chen et al., 2017). Individuals with a strong need for social connection also focus more on their role within a group and seek to maintain their ties to the community by engaging in actions beneficial to the group, such as green consumption (Do and Do, 2024).

Sports studies emphasize that sports consumption is typically associated with motives of pure entertainment (Hall and Zwarun, 2012). However, experiential sports consumption is often connected to deeper meanings, such as fostering connections between individuals (Rogers, 2018). This explains why many people consume sports even if they do not particularly enjoy it. For instance, although unfavorable game outcomes may cause negative emotions, many spectators still attend events because the experience provides an opportunity to connect with family or friends, helping to satisfy their need for social connection (Jang et al., 2017). Similarly, studies on sports event tourism highlight that these activities offer extensive social opportunities for participants, as they are often shared experiences that allow for broader social interaction during the event (Green and Jones, 2005), shifting participants' focus from competition to social engagement (Kaplanidou and Vogt, 2010).

In summary, existing literature underscores that consumers can fulfill their need for social connection through consumption choices. However, whether the need for social connection influences sports consumers' intention to engage in experiential sports consumption remains to be explored. Therefore, this study will further examine the mechanisms by which the need for social connection impacts the intention to engage in experiential sports consumption through empirical analysis.

## 3 Research hypotheses

### 3.1 Hypothesis on social exclusion and intention to engage in experiential sports consumption

Experiential sports consumption has strong social and interpersonal attributes. Its high level of social interaction and conversational value (Chanavat and Bodet, 2014) encourages individuals to share experiences (Yazici et al., 2017), making it more effective than material sports consumption in fostering social relationships, alleviating psychological crises caused by social exclusion, and enhancing subjective wellbeing (Gilovich and Gallo, 2020). For instance, experiential sports consumption behaviors such as sports tourism, spectating, and outdoor adventures are designed to encourage social connections and interpersonal interactions. Deepening these interactions helps increase interpersonal intimacy and foster positive social relationships for those who feel excluded.

Moreover, based on the theory of compensatory consumption behavior, individuals may engage in specific consumption behaviors to compensate for the psychological needs that have been threatened (Mandel et al., 2017). In the context of sports consumption, social exclusion may undermine self-concept, while participating in experiential sports can enhance self-identity, expand social networks, and restore social cohesion. In summary, experiential sports consumption can effectively foster social relationships, reduce psychological crises, and improve the wellbeing of those who experience exclusion. Thus, excluded individuals are more inclined to consume experiential sports to compensate for their threatened psychological needs. Based on this, the following hypothesis is proposed:

H1: Social exclusion has a significant positive effect on the intention to engage in experiential sports consumption.

### 3.2 The mediating role of loneliness

Loneliness is an individual's subjective perception of social isolation, typically triggered by having fewer social relationships than expected or lacking the desired level of intimacy in relationships (Gierveld and Van Tilburg, 2010). Existing studies have shown that failing to establish social connections can lead to a range of adverse psychological effects, such as loneliness, disappointment, and anxiety (Wolters et al., 2023). Wang et al. (2022) also pointed out that social exclusion easily triggers feelings of loneliness, as rejection by others disrupts an individual's expectations of interpersonal relationships, resulting in a psychological gap that intensifies feelings of loneliness (Wang et al., 2022).

Loneliness is both a direct result of social exclusion and a key driver of behavioral adjustments. It motivates individuals to escape isolation and seek social integration. Studies show that lonely consumers prefer socially oriented consumption to fulfill their interaction needs (Huang and Li, 2023). Experiential sports consumption is more effective in building positive social relationships than material sports consumption. This is because experiential sports consumption provides individuals with more opportunities to interact with others (Zhong and Mitchell, 2010) and enhances their wellbeing through these interactions (Oh et al., 2022), further reducing the harmful effects of loneliness. In summary, to mitigate the negative emotional experiences brought on by social exclusion (e.g., low self-esteem, loneliness), sports consumers may adjust their behavior and develop compensatory motivations (such as a greater intention to engage in experiential sports consumption; Adie et al., 2008) to regain healthy social relationships and achieve a more positive psychological state (Maner et al., 2007). Based on this, the following hypotheses are proposed:

H2-1: Social exclusion has a significant positive effect on loneliness.

H2-2: Loneliness has a significant positive effect on the intention to engage in experiential sports consumption.

H2-3: Loneliness mediates the relationship between social exclusion and the intention to consume experiential sports.

### 3.3 The mediating role of the need for social connection

According to the social monitoring system theory, social exclusion threatens individuals' sense of belonging, prompting them to search for cues that help them reintegrate (Pickett et al., 2004). When an individual experiences social exclusion, their sense of social connection is significantly threatened, leading to numerous adverse psychological effects. To satisfy their social needs (including self-esteem and a sense of belonging), individuals become more motivated to seek ways to establish closer ties with society (Mead et al., 2011). Research has found that when the need for social connection is unmet, individuals pay greater attention to social behaviors (Gardner et al., 2005) and are more willing to rebuild social relationships through socially-oriented consumption, thereby improving weak social connections.

In the context of sports consumption behavior, experiential sports consumption inherently possesses solid social attributes. Existing research indicates that watching sports events with friends provides enjoyable experiences and helps establish or maintain friendships and social connections, enhancing leisure quality (Zhong and Mitchell, 2010). Even when watching live sports events through social TV, the participatory experience fosters interaction, emotional exchange (Rejikumar et al., 2022), and content sharing, fulfilling the need for social connection. Additionally, a study on skiing and golf leisure activities found that consumers engage in these activities for inherent pleasure and to establish and maintain friendships and social connections, enhancing their social and leisure quality of life (Song et al., 2022). In summary, sports consumers who perceive social exclusion will likely experience a heightened need for social connection. They may engage in experiential sports consumption activities with vital social attributes to rebuild their social relationships. Based on this, the following hypotheses are proposed:

H3-1: Social exclusion has a significant positive effect on the need for social connection.

H3-2: The need for social connection has a significant positive effect on the intention to engage in experiential sports consumption.

H3-3: The need for social connection mediates the relationship between social exclusion and the intention to engage in experiential sports consumption.

### 3.4 The chain mediating role of loneliness and the need for social connection

When social exclusion occurs, individuals perceive their interactions or emotional connections with others as falling short of expectations, leading to feelings of loneliness (Wang et al., 2022). Loneliness is a subjective experience of lacking social connections, further triggering the desire to rebuild social relationships and enhancing the need for social connection (Satici et al., 2016). Loneliness not only serves as a direct result of social exclusion, but may also be an important driver of individual behavioral adjustment (Burholt et al., 2020). It provides a critical path to compensate for the psychological damage caused by social exclusion by deepening the individual's desire for social connection. According to self-determination theory, intrinsic needs drive all behavior and activity (Gilal et al., 2019). Individuals' needs shape their psychology and attitudes toward sports consumption services and directly influence their consumption processes and behaviors (Funk et al., 2012). For example, material needs reflect the value demands for the functional attributes of sports products. In contrast, the need for social connection reflects the value demands for the social qualities of sports and leisure activities (Jang et al., 2021).

Based on the theory of compensatory consumption behavior, lonely consumers driven by the desire for social connection may directly seek social interactions to alleviate their loneliness (Kim and Gal, 2014). Rippé et al. (2018) confirmed this, showing that consumers experiencing social and emotional loneliness actively seek social experiences, such as visiting brick-and-mortar stores to communicate with sales staff (Rippé et al., 2018). Self-determination theory suggests that social exclusion drives loneliness and anxiety, prompting individuals to seek substitutes for unmet needs and fostering behaviors aimed at social support and connection. Experiential sports consumption is more likely than material consumption to provide opportunities for social interaction, creating a better sense of belonging and social connection (Keyes et al., 2023). For example, for older adults, participating in local sports team events can increase emotional support and fulfill their need for belonging, thereby improving their subjective wellbeing (Inoue et al., 2020). Similarly, e-sports enthusiasts' primary reason for participating in e-sports is to positively influence existing social relationships and foster and maintain interpersonal connections within online communities (Qian et al., 2022).

In summary, social exclusion may intensify loneliness and heighten the desire for social connection. This leads to the hypothesis that consumers will be more inclined to choose experiential sports consumption for social interaction and a sense of belonging. Based on this, the following hypotheses are proposed:

H4-1: Loneliness has a significant positive effect on the need for social connection.

H4-2: Loneliness and the need for social connection mediate the relationship between social exclusion and the intention to engage in experiential sports consumption.

**Figure 1:**
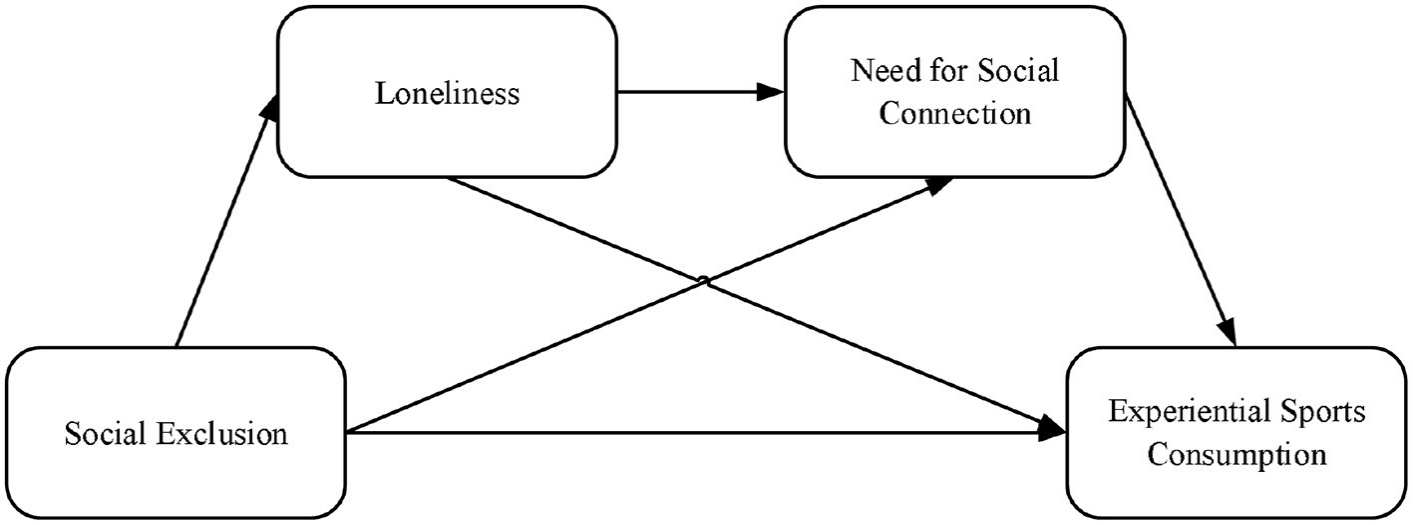
Conceptual model diagram.

In conclusion, this study develops a conceptual model of the impact of social exclusion, loneliness, and the intention to engage in experiential sports consumption. The relationships between these variables are illustrated in Figure 1.

## 4 Research design

### 4.1 Research subjects and sample

To further explore the impact of social exclusion on the intention to engage in experiential sports consumption, this study employed a survey method for data collection. To ensure the reliability and objectivity of the data, the survey emphasized that all data would be used solely for academic research purposes and that there were no right or wrong answers—respondents were encouraged to answer based on their experiences.

First, a pilot survey was conducted through an online questionnaire, with 100 questionnaires distributed and 97 returned. Of these, 89 were valid, resulting in an effective response rate of 91.75%. After statistical analysis, the reliability and validity of all scales used in the pilot survey were confirmed, allowing them to be used in the formal study.

For the formal survey, 200 paper questionnaires were distributed in person, and 300 questionnaires were distributed online, resulting in 500 questionnaires. A total of 486 responses were collected. Based on the criteria for identifying invalid questionnaires (such as excessively short response times, highly consistent answers, or missing answers), 71 invalid responses were excluded, leaving 415 valid questionnaires with an effective response rate of 85.39%. Descriptive statistics for the valid sample are provided in Table 1.

**Table 1:** Descriptive statistics of the sample.

**Statistical variable**	**Category**	**Frequency**	**Percentage (%)**
Gender	Male	200	48.2
	Female	215	51.8
Age	Under 25 years	91	21.9
	25–35 years	114	27.5
	36–45 years	113	27.2
	Over 45 years	97	23.4
Education level	High school or below	72	17.3
	Associate degree	118	28.4
	Bachelor's degree	172	41.4
	Master's degree or above	53	12.8
Monthly income	3,000 or below	91	21.9
	3,001–5,000	113	27.2
	5,001–8,000	115	27.7
	Above 8,000	96	23.1
Monthly sports consumption expenditure	200 or below	103	24.8
	200–500	122	29.4
	500–1,000	115	27.7
	Above 1,000	75	18.1

### 4.2 Research instruments

The scales used in this study were sourced from widely published and frequently utilized scales in major domestic and international journals. To ensure the scale's applicability to the sports consumption context, the research team used bidirectional translation to ensure semantic accuracy and refined the items through expert discussions, enhancing clarity and contextual relevance. The formal scale measured four key variables: social exclusion, loneliness, the need for social connection, and the intention to engage in experiential sports consumption. All items were rated on a 7-point Likert scale, where 1 indicated “strongly disagree” and 7 indicated “strongly agree.”

#### 4.2.1 Social exclusion

The measurement of social exclusion was adapted from previously developed social exclusion scales (Carter-Sowell, 2010), which include two dimensions: rejection and neglect. The scale comprises eight items, such as “During sports participation, I feel excluded by the people around me from their group.” The scale uses a 7-point Likert scale, where 1 indicates “strongly disagree” and 7 indicates “strongly agree,” with higher scores representing more significant levels of social exclusion. Confirmatory factor analysis showed that the standardized factor loadings for all items ranged between 0.790 and 0.831, indicating good structural validity for this scale. In this study, the Cronbach's α for this scale was 0.941.

#### 4.2.2 Loneliness

The measurement of loneliness was based on the ULS-8 loneliness scale developed by Russell et al. (1980) and later adapted by Hays and Dimatteo (1987). The scale includes eight items, such as “I often feel like I lack companionship.” A 7-point Likert scale was used, where 1 indicated “strongly disagree,” and 7 indicated “strongly agree,” with higher scores representing higher levels of loneliness. Confirmatory factor analysis showed that the standardized factor loadings for all items ranged from 0.747 to 0.832, indicating good structural validity. In this study, the Cronbach's α for this scale was 0.930.

#### 4.2.3 Need for social connection

The measurement of the need for social connection was adapted from the research conducted by Han et al. (2015). The scale consists of four items, such as “I really want to engage in activities with others.” A 7-point Likert scale was used, where 1 indicated “strongly disagree,” and 7 indicated “strongly agree,” with higher scores representing a more substantial need for social connection. Confirmatory factor analysis showed that the standardized factor loadings for all items ranged from 0.810 to 0.829, indicating good structural validity. In this study, the Cronbach's α for this scale was 0.891.

#### 4.2.4 Intention to engage in experiential sports consumption

The measurement of the intention to engage in experiential sports consumption was adapted from the Experiential Buying Tendency Scale (EBTS) developed by Howell et al. (2012). The scale includes four items, such as “If I had enough money, I would prefer to spend it on sports experiences (e.g., sports tourism, attending sports events) rather than purchasing tangible sports products (e.g., sportswear).” The translated items were further refined through expert discussions to enhance their relevance to the context of sports consumption. A 7-point Likert scale was used, where 1 indicated “strongly disagree” and 7 indicated “strongly agree.” Except for the reverse-scored fourth item, higher scores represented a stronger intention to engage in experiential sports consumption. Confirmatory factor analysis showed that the standardized factor loadings for all items ranged from 0.807 to 0.833, indicating good structural validity. In this study, the Cronbach's α for this scale was 0.893.

## 5 Results and analysis

### 5.1 Common method bias test

This study employed Harman's single-factor test to examine common method bias in the questionnaire data. This method integrates all questionnaire items for exploratory factor analysis to assess whether potential standard method bias affects the research results. The unrotated principal component analysis revealed four factors, with the first factor explaining 23.72% of the variance, well below the recommended threshold of 40%, indicating that the data in this study does not suffer from significant standard method bias.

### 5.2 Structural equation model testing

This study utilized AMOS 24.0 software to analyze the structural equation model. First, the model's goodness-of-fit was tested, and the results indicated a good fit, with specific values and reference standards shown in Table 2. Based on this, path analysis was conducted to evaluate the relationships between social exclusion, loneliness, the need for social connection, and the intention to engage in experiential sports consumption by examining the path coefficients between variables (see Table 3).

**Table 2:** Validation factor model fit.

**Indicators**	**χ^2^/df**	**RMR**	**GFI**	**AGFI**	**TLI**	**CFI**	**RMSEA**
Results	1.101	0.086	0.949	0.938	0.996	0.996	0.016
Standards	< 3	< 0.1	>0.9	>0.9	>0.9	>0.9	< 0.05
Situation	Fit	Fit	Fit	Fit	Fit	Fit	Fit

**Table 3:** Path analysis results.

**Hypothesis**	**Path**	**Standardized path coefficient**	***T*-value**	***P*-value**	**Test result**
H1	SE → IESC	0.246	4.550	*P* < 0.001	Supported
H2-1	SE → Lon	0.374	7.023	*P* < 0.001	Supported
H2-2	Lon → IESC	0.065	1.202	*P* = 0.229	Not supported
H3-1	SE → NSC	0.220	4.053	*P* < 0.001	Supported
H3-2	NSC → IESC	0.315	5.538	*P* < 0.001	Supported
H4-1	Lon → NSC	0.294	5.330	*P* < 0.001	Supported

SE, Social Exclusion; IESC, Intention to Engage in Experiential Sports Consumption; Lon, Loneliness; NSC, Need for Social Connection.

**Figure 2:**
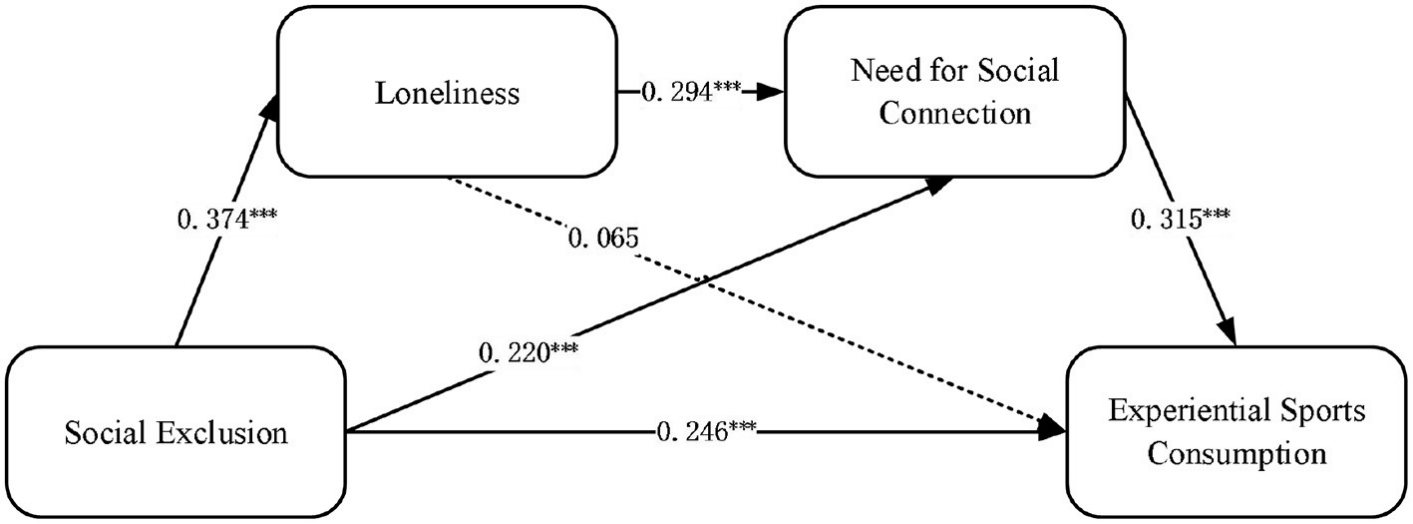
Conceptual model central path coefficient diagram. ***P < 0.001.

The main effects of this study are illustrated in Figure 2, where path analysis clearly demonstrates the significant relationships between social exclusion, loneliness, the need for social connection, and the intention to engage in experiential sports consumption. Specifically, social exclusion has a significant positive impact on the intention to engage in experiential sports consumption (γ = 0.246, *P* < 0.001), loneliness (γ = 0.374, *P* < 0.001), and the need for social connection (γ = 0.220, *P* < 0.001), confirming the validity of hypotheses H1, H2-1, and H3-1. This indicates that individuals with stronger feelings of social exclusion are more likely to engage in experiential sports consumption to alleviate negative emotions and seek psychological compensation through loneliness and the need for social connection. However, loneliness does not significantly affect the intention to engage in experiential sports consumption (γ = 0.065, *P* > 0.05), meaning hypothesis H2-2 is not supported. This suggests that while loneliness reflects an individual's lack of social connection, its direct impact on driving the intention to engage in experiential sports consumption is relatively limited. Additionally, the need for social connection significantly positively affects the intention to engage in experiential sports consumption (γ = 0.315, *P* < 0.001), supporting hypothesis H3-2. Finally, loneliness significantly impacts the need for social connection (γ = 0.294, *P* < 0.001), confirming hypothesis H4-1. This indicates that individuals with stronger feelings of loneliness are more likely to develop a need for social connection to alleviate their sense of psychological isolation.

### 5.3 Mediation effect test

To examine the mediating effects of loneliness and the need for social connection, this study employed the Bootstrap method, which is widely used in mediation analysis for its robustness and independence from the assumption of normal data distribution. Using AMOS 24.0 software, the study performed 5,000 resamples on 415 samples to ensure the estimates' stability and the confidence intervals' accuracy. In the Bootstrap analysis, a mediating effect is considered significant if the 95% confidence interval does not include 0. The results of the Bootstrap analysis for the mediation effect significance test are presented in Table 4.

**Table 4:** Mediation effect test results.

**Path**	**Effect value**	**SE**	***P*-value**	**Bias-corrected 95% CI**		**Percentile 95% CI**		**Mediation percentage (%)**
				**Lower**	**Upper**	**Lower**	**Upper**	
Total effect	0.374	0.070	*P* < 0.001	0.236	0.51	0.239	0.512	100
Direct effect	0.246	0.064	*P* < 0.001	0.131	0.382	0.13	0.38	65.78
Indirect effect	0.128	0.038	*P* < 0.001	0.062	0.212	0.058	0.207	34.22
SE → Lon → IESC	0.024	0.022	*P* = 0.192	−0.014	0.073	−0.017	0.069	6.42
SE → NSC → IESC	0.069	0.025	*P* < 0.001	0.029	0.132	0.026	0.125	18.45
SE → Lon → NSC → IESC	0.035	0.013	*P* < 0.001	0.016	0.068	0.014	0.063	9.36

SE, Social Exclusion; IESC, Intention to Engage in Experiential Sports Consumption; Lon, Loneliness; NSC, Need for Social Connection.

As shown in Table 4, the 95% confidence interval for the effect of loneliness on the relationship between social exclusion and the intention to engage in experiential sports consumption includes 0, and the *P*-value is more significant than 0.05, indicating that the mediating effect is not substantial. Therefore, hypothesis H2-3 is not supported.

In contrast, the 95% confidence interval for the effect of the need for social connection on the relationship between social exclusion and the intention to engage in experiential sports consumption does not include 0, and the *P*-value is < 0.001. This indicates that the mediating effect is significant, with a standardized effect value of 0.069, accounting for 18.45% of the total effect, thus supporting hypothesis H3-3.

Furthermore, the combined effect of loneliness and the need for social connection on the relationship between social exclusion and the intention to engage in experiential sports consumption also has a significant indirect effect, as the 95% confidence interval does not include 0 and the *P*-value is < 0.001. The standardized effect value is 0.035, accounting for 9.36% of the total effect, indicating the presence of a chain mediation effect, thereby supporting hypothesis H4-2.

## 6 Discussion

### 6.1 The relationship between social exclusion and the intention to engage in experiential sports consumption

This study found a significant positive correlation between social exclusion and the intention to engage in experiential sports consumption. Individuals experiencing social exclusion are more likely to seek experiential sports to alleviate negative emotions and rebuild social connections. This finding aligns with previous research, which concluded that experiential sports consumption can provide individuals with satisfaction and positive feelings to counteract negative moods (Armbrecht and Andersson, 2020; Guo et al., 2024; Inoue et al., 2017). However, this study reveals how social exclusion drives individuals to adopt experiential sports consumption as a proactive psychological compensation strategy to rebuild social connections and enhance their sense of belonging and self-worth.

For individuals facing social exclusion, deriving a sense of identity and fulfillment through material sports consumption is more challenging. Instead, they are more inclined to choose experiential sports consumption to mitigate negative emotions and restore a sense of value. According to compensatory consumption theory, when the self-concept or self-efficacy of sports consumers is threatened, they tend to choose consumption behaviors that address the threat and restore their self-efficacy to compensate for psychological deficiencies (Rucker and Galinsky, 2008). Furthermore, Caprariello and Reis (2013) found that consumers are likelier to share experiences than material goods. Thus, individuals excluded from social interactions during sports participation tend to choose experiential sports consumption, which provides social value to compensate for interpersonal deficits.

### 6.2 The mediating role of loneliness

This study revealed the positive effect of social exclusion on loneliness among sports consumers, indicating that the higher the experience of social exclusion, the higher the corresponding level of loneliness. This result aligns with the predictions of the Need-Threat Model (Williams, 2009), indicating that social exclusion, as an experience of interpersonal disruption, intensifies individuals' sense of social disconnection, thereby increasing feelings of loneliness (Yue et al., 2022). However, contrary to expectations, loneliness does not significantly mediate the relationship between social exclusion and the intention to engage in experiential sports consumption. Nonetheless, loneliness plays a significant role in the mediation chain involving the need for social connection.

This outcome may be due to loneliness as a negative psychological experience, exhibiting a complex bidirectional influence on behavioral choices. On the one hand, they may develop a tendency to change their weak social state by trying to establish friendships, build harmonious relationships, and seek group acceptance (DeWall et al., 2009; Wang et al., 2012). On the other hand, loneliness may reinforce individuals' memories of past interpersonal failures, leading to negative evaluations of future social interactions and fostering a tendency for self-protection (Cacioppo and Hawkey, 2009; Lemay et al., 2024).

These two conflicting tendencies shape the psychological characteristics of lonely individuals and influence their behavioral choices in specific contexts. For example, research has found that lonely individuals are more likely to form attachments to inanimate objects to avoid potential social risks. This also helps explain the findings of this study: if lonely sports consumers adopt a defensive attitude toward others, they may take a passive approach when seeking support or connection (Anderson and Martin, 1995), which results in no direct relationship between loneliness and experiential sports consumption, which inherently involves social attributes. However, when their need to establish close interpersonal relationships becomes dominant—manifested as a heightened need for social connection—lonely sports consumers are more likely to significantly influence their intention to engage in experiential sports consumption. This finding enriches the research on the pathways of loneliness in consumption behavior. Further, it suggests that the effects of loneliness may have different manifestations depending on the context and psychological needs.

### 6.3 The mediating role of the need for social connection

This study found that the need for social connection mediates the relationship between social exclusion and the intention to engage in experiential sports consumption. Specifically, social exclusion stimulates a desire for social connection, and the social interaction attributes of experiential sports consumption help individuals build positive relationships, alleviating negative emotions caused by exclusion. Consistent with previous studies, prior research has also highlighted that experiences of social exclusion can trigger a desire to reconnect with others (Maner et al., 2007), prompting individuals to adjust their behavior accordingly.

For example, studies on help-seeking behavior suggest that individuals experiencing social exclusion are more willing to seek help as rejection heightens their need for belonging (Molden et al., 2009). Such behavior helps them establish positive social connections, fulfilling their need for belonging (Addis and Mahalik, 2003). In the context of consumer behavior, research highlights that the social nature of experiential consumption fosters stronger social connections (Caprariello and Reis, 2013). In contrast, material purchases focus on individual accumulation, which may weaken social bonds (Kumar et al., 2024). Based on this theoretical perspective, this study combines social exclusion, social connection needs and experiential sport consumption to reveal further how the particular context of sport consumption can effectively fulfill individuals' social connection needs, and in the process extends the scope of application of previous studies.

### 6.4 The chain mediating role of loneliness and the need for social connection

This study found that loneliness and the need for social connection play a significant chain mediating role in the impact of social exclusion on the intention to engage in experiential sports consumption. This finding reveals the psychological mechanism by which social exclusion influences consumer behavior through loneliness and the need for social connection. Consistent with previous studies, social exclusion often threatens individuals' sense of belonging and social connection (Wesselmann et al., 2023), triggering a strong need to re-establish social ties (Lakin et al., 2008). This social motivation or need has been demonstrated to drive participation in sports consumption activities (Paek et al., 2021). For example, Kim et al. noted that interpersonal interaction with other consumers is one of the primary motivations for attending sports events (Kim et al., 2019). The present study expands on this and related research by revealing the specific role of the chain-mediated relationship between loneliness and the need for social connectedness in the context of sport consumption.

Moreover, previous research has emphasized that experiences of social isolation, including social exclusion, often intensify individuals' pursuit of materialism, reinforcing their sense of loneliness (Pieters, 2013). Materialism, as a value orientation, emphasizes the acquisition of material wealth (Dittmar and Isham, 2022; Richins and Dawson, 1992) and possessing material goods (Carter and Gilovich, 2010), and this value is usually tied to material purchases in consumer behavior (Pandelaere, 2016). However, less past research has explored how social exclusion affects experiential consumer behavior through the dual role of loneliness and the need for social connection.

The results of the chain mediation effect between loneliness and the need for social connection suggest that experiences of social exclusion amplify loneliness. The need for social connection helps activate the positive psychological need for interpersonal belonging in lonely individuals while suppressing the negative emotional tendency to avoid social risks, making them more inclined toward experiential sports consumption. Furthermore, the study indicates that loneliness cannot directly translate into an intention to engage in experiential sports consumption; it is only through the activation of the need for social connection that this transformation occurs. These insights suggest breaking the vicious cycle of loneliness and materialism, suggesting that fostering social connection can redirect lonely individuals from materialistic pursuits toward more socially enriching experiences.

## 7 Conclusion and outlook

### 7.1 Research conclusions

In summary, this study reaches the following conclusions: (1) Social exclusion significantly and positively predicts the intention to engage in experiential sports consumption. (2) Social exclusion has a significant positive impact on individuals' loneliness and need for social connection. (3) The direct effect of loneliness on the intention to engage in experiential sports consumption is not significant, nor is its mediating effect in the relationship between social exclusion and experiential sports consumption intention. (4) The need for social connection partially mediates the relationship between social exclusion and the intention to engage in experiential sports consumption. (5) A chain mediation effect exists, in which both loneliness and the need for social connection mediate the relationship between social exclusion and the intention to engage in experiential sports consumption.

### 7.2 Theoretical contributions

1. Broadening the scope of social exclusion research: Previous studies have primarily focused on the impact of social exclusion in areas such as conspicuous consumption (Lee and Shrum, 2012), impulsive buying (Mead et al., 2011), anthropomorphic consumption (Liu et al., 2022), green consumption (Guo et al., 2020), and status consumption (Walasek and Brown, 2016). This study integrates social exclusion with experiential sports consumption for the first time, showing that social exclusion influences consumption intentions through loneliness and the need for social connection. It enriches research on social exclusion and sports consumption while offering new strategies to address social exclusion.
2. This study innovatively reveals how loneliness indirectly influences experiential sports consumption through the need for social connection, clarifying their chain mediating role. The findings indicate that loneliness does not directly impact the intention to engage in experiential sports consumption but exerts an indirect effect through the need for social connection. This discovery deepens the understanding of the relationship between loneliness and consumer behavior and extends the theoretical boundaries of social exclusion and experiential consumption.
3. Shifting the focus to antecedents of experiential sports consumption: While most prior research has focused on the outcome variables of experiential sports consumption, studies on its antecedents have been relatively scarce, with most focusing on socio-economic factors such as social class and financial status. This study explores the psychological antecedents of experiential sports consumption, expanding research in this area.

### 7.3 Recommendations

Research indicates that social exclusion significantly and positively predicts the intention to consume experiential sports. Based on this, relevant sports enterprises and organizations could consider incorporating elements into event design that attract marginalized groups, such as reducing participants' sense of social isolation through social interactions and team collaboration. Particularly in specific cultural contexts, such as regions with strong local cultures or relatively underdeveloped economies, sports activities' social and inclusive nature may enhance their appeal.

This study highlights loneliness as a critical emotional factor. Sports enterprises should focus on enhancing the emotional value of experiential products by addressing psychological aspects such as enriching experiences, fostering friendships, and promoting social interactions. More socially oriented events and interactive activities, such as parent-child programs and community sports events, could be designed. For specific groups like adolescents or the elderly, strengthening group connections can effectively increase participation.

The study also finds that the need for social connection partially mediates the relationship between social exclusion and the intention to engage in experiential sports consumption. Therefore, local governments and social organizations should collaborate to implement more personalized sports service programs, especially in areas with high population mobility or groups at high risk of exclusion. For instance, promoting participation in sports tourism, fitness and leisure activities, and sports spectating can enhance experiential sports consumption. Interregional sports interaction and exchange can strengthen social connections, helping groups overcome loneliness and increasing their engagement in experiential sports consumption.

### 7.4 Research limitations and future direction

This study has several limitations, which offer opportunities for further exploration in future research: (1) This study did not differentiate between different types of social exclusion (e.g., rejection or neglect). In contrast, existing research has shown that different types of social exclusion can have varying effects on individuals' emotions (Chow et al., 2008), cognition (White and Argo, 2009), and psychological needs (Mourey et al., 2017), leading to different behaviors. Future studies should explore whether other types of social exclusion influence the research conclusions in distinct ways. (2) The present study used self-report to measure social exclusion levels, which may have been influenced by the social approval effect, limiting external validity. Future research could validate the robustness of the results and enhance the practical guidance value by manipulating social exclusion situations in the laboratory or conducting field experiments to simulate real experiences (Lee and Shrum, 2012; Sinha and Lu, 2019). (3) This study focuses on the impact of social exclusion on experiential sports consumption from the perspective of social relationships. Future research could explore the roles of variables such as wellbeing and self-concept to refine the theoretical framework further. (4) This study did not address potential biases arising from sample selection, particularly the heterogeneous effects of social exclusion on different populations. Future research should further explore these issues.

## Data Availability

The raw data supporting the conclusions of this article will be made available by the authors, without undue reservation.
